# Automatic Annotation of Narrative Radiology Reports

**DOI:** 10.3390/diagnostics10040196

**Published:** 2020-04-01

**Authors:** Ivan Krsnik, Goran Glavaš, Marina Krsnik, Damir Miletić, Ivan Štajduhar

**Affiliations:** 1 Department of Computer Engineering, Faculty of Engineering, University of Rijeka, Vukovarska 58, 51000 Rijeka, Croatia; ivankrsnik93@gmail.com; 2 School of Business Informatics and Mathematics, University of Mannheim, 68159 Mannheim, Germany; goran@informatik.uni-mannheim.de; 3 Faculty of Veterinary Medicine, University of Zagreb, Heinzelova 55, 10000 Zagreb, Croatia; mkrsnik94@gmail.com; 4 Clinical Hospital Centre Rijeka, University of Rijeka, Krešimirova 42, 51000 Rijeka, Croatia; damir.miletic@medri.uniri.hr; 5 Center for Artificial Intelligence and Cybersecurity, University of Rijeka, Radmile Matejčić 2, 51000 Rijeka, Croatia

**Keywords:** free-form radiology report, automatic labeling, decision support system, natural language processing, machine learning, word embedding, knee

## Abstract

Narrative texts in electronic health records can be efficiently utilized for building decision support systems in the clinic, only if they are correctly interpreted automatically in accordance with a specified standard. This paper tackles the problem of developing an automated method of labeling free-form radiology reports, as a precursor for building query-capable report databases in hospitals. The analyzed dataset consists of 1295 radiology reports concerning the condition of a knee, retrospectively gathered at the Clinical Hospital Centre Rijeka, Croatia. Reports were manually labeled with one or more labels from a set of 10 most commonly occurring clinical conditions. After primary preprocessing of the texts, two sets of text classification methods were compared: (1) traditional classification models—Naive Bayes (NB), Logistic Regression (LR), Support Vector Machine (SVM), and Random Forests (RF)—coupled with Bag-of-Words (BoW) features (i.e., symbolic text representation) and (2) Convolutional Neural Network (CNN) coupled with dense word vectors (i.e., word embeddings as a semantic text representation) as input features. We resorted to nested 10-fold cross-validation to evaluate the performance of competing methods using accuracy, precision, recall, and $F_{1}$ score. The CNN with semantic word representations as input yielded the overall best performance, having a micro-averaged $F_{1}$ score of 86.7%. The CNN classifier yielded particularly encouraging results for the most represented conditions: degenerative disease (95.9%), arthrosis (93.3%), and injury (89.2%). As a data-hungry deep learning model, the CNN, however, performed notably worse than the competing models on underrepresented classes with fewer training instances such as multicausal disease or metabolic disease. LR, RF, and SVM performed comparably well, with the obtained micro-averaged $F_{1}$ scores of 84.6%, 82.2%, and 82.1%, respectively.

## 1. Introduction

Medical radiology reports contain information concerning patients’ medical conditions that are most commonly recorded in a narrative form. The reports are normally established by radiologists making a visual inspection of radiology scans. Both the reports and the scans are nowadays usually stored in Picture Archiving and Communication Systems (PACS). These databases, most commonly used in hospitals worldwide, are repositories of vast amounts of information. This information is seldom accessed, once its clinical usefulness expires in regard to patient followup. One of the reasons for the poor exploitation of this already gathered information lies in the fact that the reports are normally unstructured and there exists no query-capable reliable formal connection between the established reports and the presence of a clinical condition.

There have been diverse reports on using text mining (TM) and natural language processing (NLP) techniques on electronic medical records (EMR) and similar healthcare-related information [1,2,3,4], and only a few are mentioned here (only a fraction of them deal with radiology reports). Research efforts relating to multilabel classification of clinical free text in radiology reports, using standardized labels, was reported in [5]. Through MedLEE, an NLP system, it has been shown that automated extraction of information from clinical narratives (radiology reports, discharge summaries, and other document types) is possible [4]. A good scope review into radiology report-processing efforts is also presented in [4]. A more recent work involving the use of artificial neural networks and word embeddings for automated diagnosis coding of radiology reports is reported in [6]. A study used an emergency department’s earlier medical records to predict and reduce its overcrowding [7]. A very high accuracy at classifying death certificates into four high impact diseases of interest was reported in [8]. Classification of spontaneous reports of adverse events following vaccination into potentially anaphylaxis-negative or -positive using a rule-based classifier was presented in [9]. Multiple studies demonstrated the feasibility of classifying disease outbreak reports with respect to their relevance [10,11]. A rule-based system supported by machine learning methods for detecting Framingham heart failure criteria from EMR release notes was presented in [12]. Smoke term dictionaries were trained on Amazon customer reviews to detect possible safety and efficacy concerns regarding several groups of products [13]. A modular system of pipelined components combining rule-based and machine learning techniques aiming at information extraction from the clinical narrative was introduced by [14]. Problem–action relation patterns were detected, classified, and extracted from clinical narrative texts in [15].

Clinicians are often reluctant accepting the suggestions made by a specific decision support system, if the details of the underlying automatic decision-making process are not clearly explained. Therefore, there exists a need for the so-called explainable AI, which deals with making both the learning and the inference process transparent and traceable [16]. However, to reach a level of truly trustworthy decision-supported processes in the clinic, assessing the so-called causability is needed [17]. Whereas the predictive properties of models can be assessed using standard quantitative evaluation metrics, the quality of explanations made can be evaluated using subjective measurements [18].

Building models for extracting clinical conditions from unstructured radiology reports is a necessary step for building query-capable report databases. Apart from the obvious application of such a model for querying medical records, accurate labeling of medical exams is a prerequisite for linking all the findings assigned to them to specific clinical conditions. In turn, this is also a crucial step in building a repository of labeled radiology scans. If a sufficiently accurate labeling of a large PACS archive existed, it could be used for supervised learning of an image-annotation model, outlier/error detection model, and a transfer-learning model. These are the main motives behind this work.

Although being beyond the narrow scope of this work, the reader should note that computer-aided diagnosis involving organ segmentation and detection/classification of illnesses or injuries from radiology scans (i.e., images) of the knee joint area was under consideration in several papers [19,20,21,22,23,24].

In this work, we examine the possibility of automatically establishing clinical outcomes directly from the text of free-form radiology reports recorded in a standard Croatian clinical setup. Our goal was to inspect whether more common clinical conditions (having more numerous reports) could be detected accurately and to determine the expected level of accuracy of such models. In addition, we were interested in observing the amount of data needed for training such models because performing manual data labeling can be tiresome, time-consuming, and expensive. In this case study, we concentrate on an available dataset from Clinical Hospital Centre (CHC) Rijeka, Croatia.

We analyze a total of 1295 radiology reports established from magnetic resonance imaging (MRI) scans of knees, gathered retrospectively from the PACS of CHC Rijeka. Written approval for performing this research has been obtained from the Hospital Ethics Committee, under Class 003-05/14-1/40, Reg. No. 2170-29-02/1-14-2, 30 October 2014. We manually labeled the reports for 14 most commonly reported clinical conditions: clean, unclear, arthrosis, injury, degenerative disease, inflammatory disease, neoplasm, multicausal disease, development anomaly, metabolic disease, neoplasm-like growth, idiopathic disease, autoimmune disease, and genetic disease.

With respect to automated text classification, in this work, we compared the approaches from the two main paradigms: (1) symbolic text classification, in which texts are represented with sparse vectors of TF-IDF weights, used as input features for traditional machine learning algorithms, such as Logistic Regression (LR) or Support Vector Machine (SVM); and (2) a more recent semantic text classification paradigm, in which dense semantic representations of words—word embeddings—are introduced as input to a neural architecture. Different deep learning architectures have been tried in a number of medical text classification tasks [25,26,27], including automated classification of radiology reports [6,28,29]. While recurrent [29,30] and attention-based neural networks [27,31] may present a viable solution, convolutional neural networks (CNN) seem to generally offer an edge in classification performance as well as faster training times [6,29]. Furthermore, due to their efficiency and being less data-hungry than, e.g., recurrent networks, CNNs have profiled themselves as a go-to text classification architecture in general-purpose natural language processing [32,33,34]. For these reasons, we adopt a CNN architecture for our neural classification model.

Overall, we obtained the best classification performance with the CNN model coupled with word embeddings as input. The obtained micro-average $F_{1}$ score of 86.7% is promising and renders automated classification of radiology reports in Croatian feasible, especially if more annotated reports (i.e., a larger training dataset) are to be collected in the future. Nonetheless, the symbolic classification methods (LR or SVM with sparse TF-IDF vectors as input) proved to be a more reliable solution than a CNN coupled with word embeddings for conditions (i.e., labels) with less training instances. Due to the lack of previous classification efforts for the same setting (condition classification from free-form diagnoses in Croatian language), there is no known standardized benchmark for comparison (e.g., a baseline $F_{1}$ score). This is why in this work we extensively evaluate of a number of standard and state-of-the-art text classification techniques. Henceforth, the work presented here can be used for future comparisons of predictive models built from free-form diagnoses written in Croatian, as well as in other similar Slavic languages.

The rest of the article is organized as follows. In Section 2, we present in detail the data used and explain the process of manually labeling clinical conditions. We next describe our text classification methodology: (1) text preprocessing techniques for radiology reports, (2) the chosen knowledge-representation models, along with (3) the associated supervised machine-learning algorithms and lastly (4) the experimental setup. Finally, we report and discuss the classification results in Section 3.

## 2. Materials and Methods

### 2.1. Radiology Reports and Labeling

Data were gathered retrospectively from the CHC Rijeka PACS database, as a part of another study which focused on developing an automated method for assessing anterior cruciate ligament (ACL) injuries in a human knee from MRI scans [22]. The dataset used for this research consists of 1295 unstructured radiology reports, describing the clinical condition of a knee. The reports are written in Croatian language. Each report was created by a trained radiologist in a standard clinical setup. Clinical assessment was performed by inspecting one or more scanning sequences, obtained using magnetic resonance imaging. The reports were generated between the year 2007 and 2014, based on scans performed using a Siemens Avanto 1.5T MR scanner (Rijeka, Croatia), and obtained by either T1 (≈25%), T2 (≈4%) or proton density (≈71%) weighting technique (sequences not being mutually exclusive). Details concerning the reports are described in more detail in [22].

In this study, a set of 14 most frequently occurring clinical conditions was determined. These included and were labeled as: clean, unclear, arthrosis, injury, degenerative disease, inflammatory disease, neoplasm, multicausal disease, development anomaly, metabolic disease, neoplasm-like growth, idiopathic disease, autoimmune disease, and genetic disease. The labels were set this way in order to envelop multiple clinical observations of interest for this type of diagnostic examination at the CHC Rijeka. The reader should note that the chosen labels, albeit being highly relevant for the clinical practice of CHC Rijeka, do not necessarily abide to the International Statistical Classification of Diseases and Related Health Problems (ICD-10). Reported clinical observations assigned to each label are shown in Table 1. Each radiology report was manually labeled with one or more labels by an expert radiologist.

Occurrence of a condition was modelled as a binary variable, being 1 if the condition was recognized in the report, or 0 otherwise. The appearance counts for each of the 14 conditions are also shown in Table 1. As multiple conditions may appear in the same report (i.e., we are dealing with a multi-label classification problem), the response variable was set up as a binary vector. Observation counts of label combinations present in the data are shown in Table 2.

The theoretical number of unique binary vectors equals $\approx 2^{14}$, yet only some vector occurrences were recorded in the data. These might be interesting to clinical practitioners, as they show correlation between separate clinical conditions. Four of the conditions are observed in less than 1% of the exams included in this study. Since meaningful learning-based text classification is not possible with such a small number of training instances, we discarded these conditions (neoplasm-like growth, idiopathic disease, autoimmune disease, and genetic disease) from our automated text classification experiments. As opposed to these rarely occurring conditions, the two most frequent conditions, arthrosis and injury, are found in more than half of the reports (63% and 57%, respectively).

In order to prepare the texts written in the morphologically complex Croatian language for text classification with machine learning models, we first performed some text preprocessing steps, which we describe next.

### 2.2. Data Preprocessing

Raw radiology reports used in this study consist, on average, of 897.15 (σ=306.55) letters (including punctuation and digits, but excluding whitespaces), 137.76 (σ=45.44) words, and 12.59 (σ=3.51) sentences per report (here, σ denotes the standard deviation).

Radiology reports were first stripped of punctuation and digits, and were converted to lower case. Exploratory data analysis was used to establish a list of the most common Croatian stopwords occurring throughout the reports. The list included the following words, in descending order by the number of occurrences (stopword literal translation is parenthesized): “i” (and), “u” (in), “je” (is), “se” (self), “na” (on, at), “da” (that), “za” (for), “su” (are), “a” (and), “od” (of, from), and“t” (no meaning). Each of the words from the list was then removed from the radiology reports.

Croatian is a morphologically complex language with many different forms for the same lemma (e.g., nouns have seven cases and different case suffixes in singular and plural: koljeno (knee) → koljena, koljenu, koljenom, koljenima). Given the limited number of radiology reports in our dataset, we decided to reduce the dataset vocabulary via lemmatization. We initially planned to lemmatize the corpus with MOLEX [35], a de facto standard lemmatizer for Croatian. However, we discovered that the underlying morphological dictionary of MOLEX does not cover the vast majority of the domain-specific terms from radiology reports (e.g., patelofemoralnom → patelofemoralan (patellofemoral)). Because of this, we devized our own heuristic clustering-based lemmatization algorithm, illustrated in Algorithm 1. As the majority of morphological complexity in Croatian stems from suffixation (case forms of nouns and person forms of verbs mutually differ in suffixes), we compare whether the words match in all but the last $len_{suf}$ characters. We do not cluster words shorter than $len_{tr}$ characters, as these are dominantly general terms (i.e., not domain-specific terms) which we can lemmatize with MOLEX [35]. Should the length of each of the two words in comparison be shorter than $len_{tr}+len_{suf}$, the words must match in the first $len_{tr}$ characters. The clustering itself is a simple single pass sequential procedure. Vocabulary words are placed into clusters sequentially: each word that does not match the representatives of established clusters, starts a new cluster and becomes its representative. It is important to note that our heuristic matching function based on $len_{suf}$ is transitive: if a new word matches one of the words in the cluster, it will match all of them (and none from the other clusters). We experimented with different values for $len_{suf}$ and $len_{tr}$ and have found $len_{suf}=4$, $len_{tr}=5$ to be the most robust configuration, yielding the cleanest morphological clusters. Once we obtain the clusters, for each cluster, we select the word with the highest occurrence frequency in our corpus of radiology reports as the “lemma”. Table 3 illustrates some of the obtained morphological clusters.
**Algorithm 1:***ClusterForms*(vocab, $len_{tr}$, $len_{suf}$)Clusters←⌀ **for***w* in vocab    **if**
$len\left(w\right)>len_{tr}$        placed←False        **for**
*k* in Clusters              $len_{match}$ = $max(len_{tr},len\left(w\right)-len_{suf},len\left(k\right)-len_{suf})$              **if**
$substr\left(w,len_{match}\right)=substr\left(k,len_{match}\right)$                  Clusters[k]←Clusters[k]∪{w}                  placed=True              **if**
placed=False                  Clusters[w]←{w} **return**
Clusters

### 2.3. Symbolic Text Classification

Traditional approaches to text classification can be seen as symbolic, since they treat every word as a different symbol and establish no relations between words with respect to their actual meaning. In this paradigm, documents are represented as bags of words (BoW), that is, sparse vectors of vocabulary terms (each dimension of the vector corresponds to one vocabulary term). Following standard practice [36,37], we adopt the TF-IDF weighting scheme for BoW vectors: the value of each term appearing in the text is set to the product of the term’s frequency in the document (TF component) and inverse of the number of documents in the collection in which the term appears (IDF component). TF-IDF weighted BoW vectors of documents can now be fed as feature vectors to machine learning algorithms that require strictly numeric features as input, such as logistic regression (LR) or support vector machine (SVM). As common text classification baselines, we also experiment with two other supervised algorithms, which, albeit operating on a symbolic representation of text (no modelling of word meaning), do not require numeric vector-based representations at input: Naïve Bayes (NB) and Random Forests (RF). We next briefly describe each of these four algorithms for symbolic text classification which we evaluated in our experiments.

#### 2.3.1. Logistic Regression

Logistic regression is a simple linear discriminative binary classification model (i.e., it finds a hyperplane that aims to separate the data instances of two classes). The predictions are made by applying the logistic function on the dot product of the features $x=[x_{0},x_{1},\ldots ,x_{n}]$ and model’s weights (i.e., parameters) $w=[w_{0},w_{1},\ldots ,w_{n}]$: $h\left(x|w\right)=\sigma \left(w^{T}x\right)=1/\left(1+\exp\left(-w^{T}x\right)\right)$. The parameters w are optimized by minimising the following *cross-entropy loss* on the training set $D={\left\{x_{i},y_{i}\right\}}_{i=1}^{N}$:

$$\begin{array}{cc} J_{CE}\left(D|w\right) & =\sum_{i=1}^{N}y_{i}\;\cdot \;\ln\left(h\left(x_{i}|w\right)\right)\\ \; & +\sum_{i=1}^{N}\left(1-y_{i}\right)\;\cdot \;\ln\left(1-h\left(x_{i}|w\right)\right) \end{array}$$

Since the equation $\nabla_{w}J_{CE}\left(D|w\right)=0$ does not have a closed-form solution, the parameters of the logistic regression model are learned via numeric optimization, most often using the (stochastic) gradient descent algorithm. The cross-entropy loss is often augmented with the regularization component λ∥w∥, i.e., $J\left(D|w\right)=J_{CE}\left(D|w\right)+\lambda ∥w∥$, which serves to prevent overfitting (i.e., prevents parameter w to take overly large values) on small datasets. The regularization factor λ is then a hyperparameter of the model which needs to be optimized via cross-validation.

#### 2.3.2. Support Vector Machines

Support vector machine (SVM) is a discriminative binary classification model [38]. In its base form (i.e., without applying the kernel trick, with which the original feature space can be projected to a new higher-dimensional space), SVM is also a linear model, aiming to find the optimal weights w that multiply the features x (i.e., $w^{T}x$) in order to separate the instances of two classes. However, SVM optimizes a different objective than the logistic regression (which minimizes the cross-entropy error): it attempts to find a discriminating hyperplane that maximizes the minimal distance between the hyperplane and the closest instances (called support vectors) of the two classes, i.e.:

$$J_{SVM}\left(D|w\right)=\max\frac{1}{∥w∥}min_{i}\left\{y_{i}\;\cdot \;w^{T}x_{i}\right\}$$

Given that (1) the maximum of $\frac{1}{∥w∥}$ is the same as the minimum of ${∥w∥}^{2}$; and that (2) the above objective has additional inequality constraints corresponding to the requirement that the examples are on the correct side of the separating hyperplane ($y_{i}\;\cdot \;w^{T}x_{i}\geq 0$, for each training example $\left(x_{i},y_{i}\right)$); the optimization problem belongs to the family of quadratically constrained quadratic programming (QCQP) problems and is solved via Lagrangian multiplicators and quadratic optimization algorithms like sequential minimal optimization (SMO) [39]. The objective is augmented with the additional penalty terms for instances on the wrong side of the separating hyperplane $w^{T}x$ and the amount of penalty is regulated with the hyperparameter *C*, which needs to be optimized via cross-validation.

SVMs are famous for the so-called kernel trick with which the input features vectors x are effectively projected to a higher-dimensional space without the explicit computation of the projection. The trick is based on the kernel function $\kappa (x,x^{\prime })$ which measures the similarities between feature vectors of the training set. If the Gram matrix K corresponding to the kernel function κ—a matrix with elements being kernel function values for pairs of training instances from *D*, i.e., $K_{ij}=\kappa \left(x_{i},x_{j}\right)$—is positive semi-definite (for any *D*), than there is a guarantee that the kernel similarity $\kappa (x_{i},x_{j})$ corresponds to a dot product of the $x_{i}$, $x_{j}$ projected to some higher-dimensional space, $\kappa \left(x_{i},x_{j}\right)=\phi {\left(x_{i}\right)}^{T}\phi \left(x_{j}\right)$. According to Cover’s theorem, the set of instances of different classes are more likely to be linearly separable in the higher-dimensional space: this is why the SVM’s kernel trick is very useful for problems where the instances are nonlinearly separable in the original feature space. However, text classification is, in most cases, not one of such problems—the feature space (i.e., the size of the vocabulary) is often much larger than the number of training instances, making the instances often close to linearly separable already in the original feature space. This is why the linear SVM commonly performs better than kernel-based SVMs for text classification. We confirmed this in our experiments as well—we thus report only the results obtained with the linear SVM.

#### 2.3.3. Random Forests

Random forests [40] is an ensemble model made up of many decision trees. Ensemble models increase accuracy and decrease variance by averaging multiple predictions. Random forests is also a refinement of a previous ensemble technique called bagging. In bagging, the original dataset is resampled with replacement multiple times in order to create differing data subsets. A uniform base model is then trained on each of these subsets.

Random forests expands on the idea of bagging by varying not only data samples, but also features used for training each of the base models. Randomness can be increased further by varying the parameters of the base models. Each decision tree is built by using a randomly chosen subset of features on a randomly chosen subset of data.

The model takes a prediction from all of the trees in the forest. The final output is then either the average (in a regression problem), or the majority vote (in a classification problem) of these predictions.

#### 2.3.4. Naïve Bayes

Naïve Bayes classifier [41] is a simple generative model based on the Bayes’ theorem. It assumes that the features are conditionally independent given the class label. Depending on the assumptions made on distributions of features, there are multiple models of the Naïve Bayes classifier. Gaussian Naïve Bayes is typically used for continuous features, while multinomial and Bernoulli Naïve Bayes models are popular for discrete features.

Naïve Bayes classifier has worked quite well in many real-world situations, even in cases where the conditional independence assumption clearly does not hold. It has been particularly effective with high-dimensional sparse data regularly seen in text classification. It is often used as a baseline model in text classification and is expected to perform somewhat worse than more complex models like logistic regression and (linear) SVM [42,43].

### 2.4. Semantic Text Classification

Sparse symbolic text representations (i.e., BoW) do not capture the meaning of words: they just treat any two different words as different (and mutually equidistant) symbols. Semantic representations of words and larger units of text aim to capture the meaning of text, beyond the surface symbols, in the form of dense numeric vectors, often called embeddings. Modern word and sentence embeddings are obtained by exploiting the distributional word co-occurrence information in large corpora [44,45,46,47,48].

Dense semantic representations of text, dominantly word embeddings, are commonly fed as input features into (deep) neural networks. Convolutional neural networks (CNNs), originating from the work in computer vision [49,50], have been shown to perform particularly well on various text classification tasks [32,34]. In what follows, we first briefly describe the Continuous-Bag-of-Words (CBOW) algorithm [44] with which we pre-trained word embeddings for Croatian, and then describe the CNN-based text classification with word embeddings as input.

#### 2.4.1. Word Embeddings

Word embeddings are dense semantic vectors that encode the meaning of words from their co-occurrences in a large corpus. Let *V* be the size of the vocabulary of some large corpus and let the $w_{c-k}\ldots w_{c-1}w_{c}w_{c+1}\ldots w_{c+k}$ denote a sequence of 2k+1 words from the corpus, referred to as one *context*. Each vocabulary word is initially assigned two dense, randomly initialized *d*-dimensional vectors (d≪V): a *center vector* of the word (denoted $w_{j}$ for the *j*-th vocabulary word) and a *context vector* of the word (denoted $w_{j}^{\prime }$ for the *j*-th vocabulary word). Let $W\in R^{d\times V}$ be the matrix consisting of context vectors of all vocabulary words (each in one column) and let $W^{\prime }\in R^{V\times d}$ be the corresponding matrix of center vectors of words (each in one row).

The input to the CBOW model is the sparse *few-hot encoding* of the context without the center word, i.e., it is a sparse binary *V*-dimensional vector $x_{ctx}$ with only the elements corresponding to context words $w_{c-k}\ldots w_{c-1}$, $w_{c+1}\ldots w_{c+k}$ set to the value 1. The CBOW model is a shallow neural network which first compresses the sparse representation of the context into a dense *d*-dimensional vector, using the parameter matrix W, i.e., $h=Wx_{ctx}$, and then tries to reconstruct the sparse one-hot encoding of the center word via $W^{\prime }$, i.e., the prediction of the center word’s vector is given with ${\hat{x}}_{cnt}=W^{\prime }\;\cdot \;h=W^{\prime }\;\cdot \;\left(W\;\cdot \;x_{ctx}\right)$. Matrices W and $W^{\prime }$ are CBOW’s parameters which are optimized by minimizing the distance between the predicted vector ${\hat{x}}_{cnt}$ and the true one-hot encoding of the center word $x_{cnt}$, a binary vector with only the element at the index corresponding to the center word being set to 1.

The CBOW model is trained on all contexts from a large corpus of text: once the training is over, the dense word representations of words, i.e., the word embeddings, are stored in matrices W and $W^{\prime }$. The word embedding of the *j*-th vocabulary word is obtained as the concatenation of the *j*-th column of W and the *j*-th row of $W^{\prime }$.

We pre-train the word embeddings for Croatian by running the CBOW model (with the window size k=5) on the hrWaC corpus [51], which is, to the best of our knowledge, the largest publicly available corpus of text in Croatian language.

#### 2.4.2. Convolutional Neural Network

**Figure 1:**
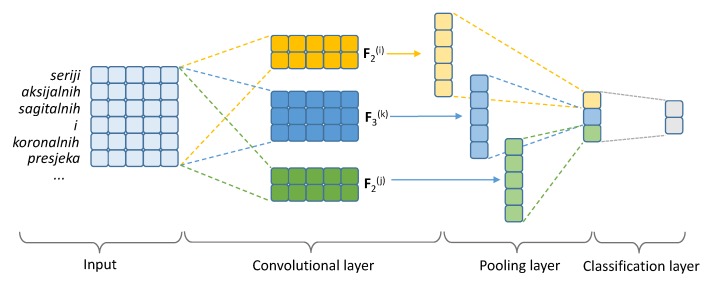
An illustration of the architecture of the vanilla CNN we used for text classification.

The illustration of the vanilla (single-layer) CNN used in our experiments is shown in Figure 1.

Let us denote the input matrix for the CNN—containing stacked pretrained word embeddings of words in the text—with $X\in R^{N\times d}$, where *N* is the length of the word sequence and *d* is the size of the pretrained word embeddings. The parameters of the CNN are the so-called *filter matrices*, sometimes also referred to as *kernels* or *kernel matrices*, $F_{s}^{\left(k\right)}\in R^{s\times d}$, where *s* is the size of the filter and *k* the index of the filter of size *s* (there are usually multiple different filters of the same size), with which we stride over the input matrix and compute convolutions. Let $X\left[n:n+s\right]\in R^{s\times d}$ be the slice of the input matrix of the size *s* that matches in size some filter $F_{s}^{\left(k\right)}$. The convolution between the input slice and the filter is then simply a sum of the element-wise multiplications between these two matrices:

$$\begin{array}{cc} \mathrm{conv}(X[n:n+s], & F_{s}^{\left(k\right)})=\\ & \sum_{i=1}^{d}\sum_{j=1}^{s}X{\left[n:n+s\right]}_{ij}\;\cdot \;{\left[F_{s}^{\left(k\right)}\right]}_{ij} \end{array}$$

A convolution is computed between a filter $F_{s}^{\left(k\right)}$ and each *s*-sized slice of the input matrix X which yields a resulting vector $x_{s}^{\left(k\right)}$ of the size N−s+1 (each convolution yields one element of that vector).

Each vector $x_{s}^{\left(k\right)}$, a result of the interaction between input X and the filter $F_{s}^{\left(k\right)}$, is then forwarded to the *pooling layer* in which the vector values are compressed into one scalar value. We use 1-max pooling, and select the largest value from $x_{s}^{\left(k\right)}$ as a feature for the final classification layer. With 1-max pooling, each filter yields one feature for the final representation vector, which we denote x. The final feature vector is then fed to a softmax regression classifier to obtain the class probabilities:

$$\hat{y}=softmax\left(xW_{cl}+b_{cl}\right)$$

with $W_{cl}\in R^{f\times 2}$ and $b_{cl}\in R^{2}$ (where *f* is the total number of convolutional filters in the model) as the parameters of the classifier.

The parameters of the convolutional text classifier described above that need to be learned on the training set are all of the filter matrices $F_{s}^{\left(k\right)}$ and the classifier parameters $W_{cl}$ and $b_{cl}$. We optimize the parameters by minimizing the cross-entropy loss (see Section 2.3.1) on the training instances. The number of convolutional filters and their respective sizes are hyperparameters of the model which we tune via cross-validation.

### 2.5. Experimental Setup and Model Evaluation

In order to allow for a fair comparison of performance of the presented machine learning models for text classification, we tune their hyperparameters and evaluate their performance via nested *K*-fold cross-validation (CV). For each of the five machine learning models, we define the set of hyperparameter configurations to be evaluated. For the outer loop of the nested *K*-fold cross-validation, we create *K* different training-test splits: for each of these splits, we perform an inner *K*-fold cross-validation in which we use *K* train-validation splits to find the optimal hyperparameter configuration; we then train the model with the whole training set of the outer loop split and evaluate its performance on the test set of the outer CV loop; finally, we report the average performance over the test sets of all outer CV folds. We set K=10 for both the outer and inner loop of our nested folded CV evaluation.

## 3. Results and Discussion

The summary of the results for all five classification models (NB, RF, LR, SVM, CNN) for the 10 knee condition labels with more than 10 instances in the dataset are shown, in terms of the $F_{1}$ score, in Table 4. In Table 5, we report more detailed results, using other evaluation metrics as well, i.e., classification accuracy, precision, and recall.

A larger discrepancy between the values of presented evaluation metrics, especially when comparing the precision–recall pair against the classification accuracy, shown in Table 5, stems directly from class imbalance (Table 1). Namely, the proportion of positive cases is rather small for all labels except arthrosis and injury. Unlike classification accuracy, which is calculated as the total unweighted proportion of misclassified instances, precision and recall are calculated as the proportions of true positive instances, against the number of instances predicted as positive and the number of instances that are observed positive, respectively. Hence, the large number of true negatives seemingly increases overall model performance in terms of classification accuracy.

Overall, the CNN model with pretrained Croatian word embeddings as input displayed the strongest performance, when micro-averaging the performance over all instances, instead of classes. As a data hungry neural model, the CNN, expectedly, performs best for the classes that have the largest number of positive instances: arthrosis, injury, and degenerative disease. The CNN, also quite expectedly, was unable to learn anything meaningful for classes with very limited number of positive instances (multicausal dis., developmental an., metabolic dis.). This is directly caused by the outlook of the loss function used for model training, where both the positive and the negative instances are treated equally, thus skewing model predictions in favor of the majority class, which becomes more apparent when dealing with classes having significantly smaller proportions of positives. LR and RF seem to be the most robust choice for these classes with very few positive instances, which also results in the best macro-averaged (average of class performances) $F_{1}$ performance. As an ensemble of decision tree classifiers, built from randomly sampled subsets of variables, RFs are capable of accurately capturing the underlying distribution (low bias) while reducing the variance, which probably explains their excellent performance on underrepresented classes. Overall, the trends in results are reasonably within expectation, with NB offering the lowest performance, and LR and CNN being the most competitive: CNN is a better choice for sufficiently large classes, where LR renders itself as the most robust choice for underrepresented classes having fewer instances. The lower complexity of the LR model, i.e., a smaller number of modelling parameters, compared to the CNN model, is the probable cause for this.

While the number of positive training instances has a key effect on the models’ quality and performance, it is worth noticing that RF, LR, and CNN achieve the best performance for the class degenerative disease, even though for this class we have a total of only 160 instances, which is approximately five times less than for the classes arthrosis and injury, for which these models obtain a slightly lower performance. This is because of a smaller variability of the language used for the class degenerative disease and some very discriminative words (e.g., osteoarthritis appears in most reports labeled with degenerative disease class, whereas very few reports of the same class do not contain it). The variability of the language among the instances of the two larger classes—arthrosis and injury—is somewhat larger.

To summarize, in this paper, we deal with the problem of automatically assigning labels to medical reports, for the purpose of creating a query-capable repository of medical records. We inspect in detail the case of radiology reports generated from MRI scans of knees, gathered retrospectively from the CHC Rijeka PACS database.

We present a thorough performance comparison of several traditional machine learning models for text classification, including logistic regression (LR), support vector machine (SVM), and random forests (RF), against the state-of-the-art text classification model based on word embeddings and convolutional neural network (CNN). We report the results obtained via nested 10-fold cross-validation. Our results, presented in Section 3, suggest that the CNN-based classifier with pretrained word embeddings as input outperforms all other models for larger classes with more positive diagnosis (i.e., text) instances. On the other hand, our experiments identify LR as the most robust choice for small classes with limited numbers of training instances. With regard to model performance in detecting individual clinical conditions, expectedly, the conditions appearing more frequently throughout the reports receive more accurate predictions.

To conclude, although our work focuses on radiology reports for knee conditions, written in the (morphologically complex) Croatian language, we believe that our study offers useful insights into the suitability of different text classification models for classifying medical reports in general, especially with respect to the size of the available annotated data.

## Figures and Tables

**Table 1:** Number of occurrences (counts) of distinct clinical conditions and examples of phrases indicating the clinical conditions. These phrases were the most frequently occurring in the used collection of unstructured radiology reports.

Clinical Condition	Count	Observation
arthrosis	820	grade I degenerative changes, chondromalacia, pseudocyst, popliteal cyst
injury	738	patella fracture, partial ACL rupture, sprain, meniscus tear, contusion
degenerative disease	160	osteoarthritis
clean	79	lateral meniscus intact, cartilage preserved, patella positioned normally
inflammatory disease	76	osteomyelitis, septic arthritis, chronic enthesitis, Osgood–Schlatter disease, rheumatoid arthritis, discoid meniscus
neoplasm	68	sessile osteochondroma, hemangioma, enchondroma, fibroma
unclear	42	patchy areas of edema, patellar tilt
multicausal disease	32	varicose veins, chondrocalcinosis, osteochondromatosis
developmental anomaly	16	tibial developmental defect, bipartite patella, knee recurvation
metabolic disease	14	osteoporosis
neoplasm-like growth	7	fibrous cortical defect
idiopathic disease	3	ACL mucoid degeneration
autoimmune disease	2	Henoch–Schönlein purpura
genetic disease	1	osteopetrosis

**Table 2:** Observation counts for each unique set of clinical conditions appearing in the radiology reports used for this research. Sets of clinical conditions are shown in descending order with respect to their observation count.

Clinical Conditions	Count	Clinical Conditions	Count
arthrosis, injury	306	arthrosis, injury, degenerative dis., neoplasm	2
injury	281	arthrosis, injury, degenerative dis., inflammatory dis.	2
arthrosis	261	arthrosis, injury, degenerative dis., multicausal dis.	2
clean	79	arthrosis, injury, degenerative dis., developmental anomaly	1
arthrosis, injury, degenerative dis.	66	arthrosis, injury, neoplasm, metabolic disease	1
arthrosis, degenerative dis.	52	degenerative dis., developmental anomaly	1
unclear	41	arthrosis, developmental anomaly	1
arthrosis, neoplasm	20	arthrosis, injury, degenerative dis., inflammatory dis., developmental anomaly, idiopathic dis.	1
arthrosis, inflammatory dis.	20	developmental anomaly	1
neoplasm	19	arthrosis, genetic dis.	1
inflammatory dis.	15	arthrosis, degenerative dis., inflammatory dis., neoplasm	1
arthrosis, injury, inflammatory dis.	15	arthrosis, injury, degenerative dis., neoplasm-like growth	1
arthrosis, degenerative dis., inflammatory dis.	11	arthrosis, degenerative dis., inflammatory dis., multicausal dis.	1
injury, neoplasm	9	arthrosis, injury, multicausal dis., idiopathic dis.	1
arthrosis, injury, multicausal dis.	9	injury, neoplasm, developmental anomaly	1
arthrosis, injury, neoplasm	7	arthrosis, idiopathic dis.	1
arthrosis, multicausal dis.	6	injury, metabolic disease	1
injury, degenerative dis.	6	neoplasm-like growth	1
arthrosis, injury, metabolic disease	5	arthrosis, inflammatory dis., developmental anomaly	1
arthrosis, injury, developmental anomaly	5	arthrosis, degenerative dis., inflammatory dis., developmental anomaly	1
injury, inflammatory dis.	4	injury, neoplasm-like growth	1
arthrosis, metabolic disease	4	degenerative dis.	1
arthrosis, degenerative dis., multicausal dis.	4	unclear, neoplasm, neoplasm-like growth	1
arthrosis, injury, degenerative dis., metabolic disease	3	arthrosis, injury, neoplasm-like growth	1
arthrosis, degenerative dis., neoplasm	3	neoplasm-like growth, autoimmune dis.	1
injury, developmental anomaly	3	autoimmune dis.	1
arthrosis, inflammatory dis., neoplasm	3	multicausal dis., neoplasm-like growth	1
injury, multicausal dis.	3	inflammatory dis., multicausal dis.	1
multicausal dis.	3	arthrosis, injury, degenerative dis., neoplasm, multicausal dis.	1

**Table 3:** Examples of morphological clusters obtained via our simple heuristic word clustering algorithm. The numbers in brackets indicate the number of occurrences in our corpus of radiology reports. Selected lemma of each cluster is emphasized.

uznapredovala (10), uznapredovalog (13), uznapredovalih (17), uznapredovalnim (1), uznapredovalim (5), uznapredovalu (2), **uznapredovale (74)**, uznapredovalosti (1) uznapredovalom (5), uznapredovali (32), uznapredovao (1)
posteromedijalni (1), posteromedijalnog (1), **posteromedijalno (9)**, posteromedijalnome (2)
infrapatelarnoj (1), infrapatelarno (29), infrapatelarnom (3), infrapatelano (1), **infrapatelarnog (33)**, infrapatelarne (1), infrapatelanog (3)
ekstenzorni (1), ekstenzije (1), ekstenzora (1), ekstenziji (1), ekstenzornom (1), ekstenzivne (1), ekstenzija (1), ekstenzorne (1), ekstenzivan (1), ekstenzivno (2), **ekstenzijom (5)**

**Table 4:** Results (in terms of $F_{1}$ scores) for all five text classification models used on each of the 10 knee condition labels. The last two rows show the micro- and macro-averaged $F_{1}$ performance over all 10 classes. The results presented here were obtained via nested 10-fold CV.

Class	NB	RF	LR	SVM	CNN
clean	24.3	25.3	47.4	**57.1**	39.5
unclear	8.7	14.8	7.5	8.7	4.5
arthrosis	83.5	89.3	89.0	88.9	**93.3**
injury	81.3	82.3	88.3	87.7	**89.2**
degenerative dis.	26.4	94.5	94.8	72.5	**95.9**
inflammatory dis.	7.1	65.6	**67.7**	36.4	52.3
neoplasm	16.2	46.0	55.4	51.6	**75.9**
multicausal dis.	0.0	27.9	**34.1**	6.1	0.0
developmental an.	0.0	31.6	**38.1**	0.0	0.0
metabolic dis.	0.0	**69.6**	**69.6**	0.0	13.3
Macro avg.	24.8	54.7	**59.2**	40.9	46.4
Micro avg.	73.8	82.2	84.6	82.1	**86.7**

**Table 5:** Detailed results (classification accuracy, precision, recall, $F_{1}$ score) for NB, RF, LR, SVM, and CNN models for the 10 knee condition labels. The last two columns show the micro- and macro-averaged performance on the selected evaluation metric over all 10 classes. The results are obtained via nested 10-fold CV.

Model	Class											
	Clean	Unclear	Arthrosis	Injury	Degenerative Dis.	Inflammatory Dis.	Neoplasm	Multicausal Dis.	Developmental An.	Metabolic Dis.	Macro Average	Micro Average
*Classification accuracy*												
NB	95.7	96.8	75.2	75.5	87.5	93.9	95.2	97.5	98.8	98.9	91.5	91.5
RF	95.0	96.5	86.2	79.7	98.7	96.7	95.8	97.6	99.0	99.5	94.5	94.4
LR	96.1	96.2	85.5	86.3	98.8	96.8	96.2	97.9	99.0	99.5	95.2	95.2
SVM	96.8	96.8	85.0	85.3	94.2	95.2	96.5	97.6	98.8	98.9	94.5	94.5
CNN	96.0	96.8	91.0	87.2	99.0	95.9	97.8	97.4	98.8	99.0	95.9	95.9
*Precision*												
NB	90.0	66.7	74.4	74.5	48.3	37.5	100	0.0	0.0	0.0	49.1	73.7
RF	47.8	36.4	91.7	85.3	98.0	85.4	71.9	54.5	100	88.9	76.0	89.0
LR	69.7	20.0	88.9	89.7	98.0	86.0	70.5	77.8	80.0	88.9	77.0	88.7
SVM	82.4	66.7	86.8	87.1	87.6	81.8	96.0	100	0.0	0.0	68.8	87.0
CNN	77.3	100	91.6	89.5	97.4	85.3	91.7	0.0	0.0	100	63.3	90.8
*Recall*												
NB	14.1	4.7	95.2	89.5	18.1	3.9	8.8	0.0	0.0	0.0	23.4	74.0
RF	17.2	9.3	86.9	79.5	91.3	53.2	33.8	18.8	18.8	57.1	46.6	76.4
LR	35.9	4.7	89.1	86.9	91.9	55.8	45.6	21.9	25.0	57.2	51.4	80.8
SVM	43.8	4.7	91.0	88.3	61.9	23.4	35.3	3.1	0.0	0.0	35.2	77.7
CNN	26.6	2.3	95.1	89.0	94.4	37.7	64.7	0.0	0.0	7.1	41.7	83.0
$F_{1}$ *score*												
NB	24.3	8.7	83.5	81.3	26.4	7.1	16.2	0.0	0.0	0.0	24.8	73.8
RF	25.3	**14.8**	89.3	82.3	94.5	65.6	46.0	27.9	31.6	**69.6**	54.7	82.2
LR	47.4	7.5	89.0	88.3	94.8	**67.7**	55.4	**34.1**	**38.1**	**69.6**	**59.2**	84.6
SVM	57.1	8.7	88.9	87.7	72.5	36.4	51.6	6.1	0.0	0.0	40.9	82.1
CNN	39.5	4.5	**93.3**	**89.2**	**95.9**	52.3	**75.9**	0.0	0.0	13.3	46.4	**86.7**
